# Evaluation of Citizen Epidemic Prevention Information Literacy in the Post-Epidemic Era in Mainland China

**DOI:** 10.3390/ijerph20065226

**Published:** 2023-03-22

**Authors:** Quanyong Yi, Xu Liu, Shanshan Ding, Xinyue Yao, Lisha Luo

**Affiliations:** 1 Faculty of Education, Southwest University, Chongqing 400715, China; 2 Faculty of Education, East China Normal University, Shanghai 200062, China; 3 Faculty of Education, Beijing Normal University, Beijing 100875, China

**Keywords:** epidemic prevention information literacy, health information literacy, public health crisis, evaluation index

## Abstract

Improving citizen epidemic prevention information literacy is one of the most cost-efficient and important measures to improve people’s epidemic prevention abilities to effectively deal with future public health crises. Epidemic prevention information literacy is beneficial to improve individuals’ ability to deal with public health crises in the future. By summarizing related domestic and international research, and utilizing an empirical methodology, we constructed an epidemic prevention information literacy assessment model with good reliability, validity, and model fit. The model is composed of four indicators: (1) “epidemic prevention information awareness”; (2) “epidemic prevention information knowledge”; (3) “epidemic prevention information ability”; (4) “epidemic prevention information morality”. We used the model to assess the epidemic prevention information literacy of Chinese citizens. The results showed the following: (1) the overall level of the epidemic prevention information literacy of Chinese citizens was comparatively high, however, its development was unbalanced, and the capability and moral levels of the epidemic prevention information were comparatively low; (2) the four dimensions of the epidemic prevention information literacy were different in terms of the citizens’ education levels and locations. We analyzed the probable causes of these problems, and we propose specific corresponding countermeasures. The research provides a set of methods and norms for the evaluation of citizen epidemic prevention information literacy in the post-epidemic era.

## 1. Introduction

The history of human development is also a history of the tenacious struggle against epidemic diseases. An outbreak of smallpox was reported in ancient Greece in 429 BC that led to the deaths of nearly 50% of the population of Athens. Plagues broke out many times during the Roman Empire, which is one of the reasons for its eventual decline. With the convenience of transportation and the rapid development of science and technology, globalization continues to deepen, and cross-regional and cross-country exchanges have become more frequent, which indirectly provides a convenient channel for the rapid spread of epidemic diseases. In recent years, there have been several major outbreaks, such as severe acute respiratory syndrome (SARS), influenza A (H1N1), MERS virus, Ebola virus, etc., which have caused panic in international and Chinese societies. In particular, since December 2019, we have witnessed the global outbreak of a novel coronavirus pneumonia (hereinafter referred to as “COVID-19”). So far, according to the data of the World Health Organization (WHO), the total number of confirmed cases in the world has exceeded 580 million, and the cumulative death toll is more than 6 million. COVID-19 has caused immeasurable losses to China and the international economy and society.

In the post-epidemic era, epidemic prevention is a national campaign, and it is a war for people at the macro level with the construction of prevention and control mechanisms under the guidance of national governance systems, and at the micro level with the participation and self-protection of every citizen. In big data era, information plays an increasingly important role in citizen epidemic prevention; however, citizen epidemic prevention literacy is also facing great challenges, which are mainly reflected in the sharp increase in the amount of information, the vague authority of information sources, and the lack of ability to obtain and identify massive amounts of information, which greatly affect the epidemic prevention behaviors of citizens.

Studies have proved that people who have a high level of knowledge of epidemic prevention and health are better able to comply with the behavioral measures of epidemic prevention and have a more positive attitude towards the disease [1]. Amidst the growing technological and informational dissemination during the COVID-19 pandemic, an inadequate level of health literacy can expose patients to misunderstanding, especially in communication networks, such as the internet, which exhibit both facts and untruths about a wide variety of subjects [2]. As the most populous and developing country in the world, China faces unimaginable difficulties and problems in epidemic prevention. Therefore, the investigation of information literacy in epidemic prevention in China can provide reference for international epidemic prevention. In this context, epidemic prevention information literacy has become one of the key points to improve the epidemic prevention abilities of citizens, and it plays an immeasurably important role in epidemic prevention and control in the post-epidemic era. The epidemic situation of novel coronavirus pneumonia has exposed the relatively low quality of the information literacy of Chinese citizens in epidemic prevention. For example, the public cannot obtain or utilize information through authoritative channels to guide their own epidemic prevention and protection. The public’s ability to judge and screen information is insufficient, which has led to the dissemination of false epidemic information. During the period of epidemic prevention and control, rumors spread in an endless stream, and they even led to the “information epidemic” [3]. In order to conduct their own epidemic prevention work, the key for citizens is to improve their epidemic prevention information literacy. Only with good epidemic prevention information literacy can we rationally and scientifically deal with all kinds of information in the post-epidemic era, and scientifically and effectively perform our own epidemic prevention and protection work. Epidemic prevention information literacy is one of the most fundamental, economic, and effective measures to improve the epidemic prevention abilities of citizens. The general public needs information literacy skills to enable them to better manage the COVID-19 crisis and similar health-related crises that they will be faced with in the future. In this pandemic, it is essential that related agents began to enhance the health information literacy of the general public as immediate action is required in this regard [4]. This study aims to investigate the information literacy of epidemic prevention in mainland China by constructing an epidemic prevention information measurement model and developing a tool with high reliability and validity. The international significance of Chinese research in the fields of epidemic prevention information literacy include raising public awareness of prevention and control measures, providing a reference for other countries, and promoting the development of global health security.

In this study, we focused on the long-term development of the country. This epidemic should be taken as an opportunity to promote the cultivation of citizen epidemic prevention information literacy, and to explore its important role in the post-epidemic era to provide guidance for the world to win this major epidemic battle as soon as possible, and to effectively deal with future public health crises.

## 2. Literature Review

### 2.1. Research on Connotation of Epidemic Prevention Information Literacy

The concept of epidemic prevention information literacy is rare in China and worldwide. Most scholars think of it is a part of health information literacy. In 1995, the national health education standard of the United States started from information literacy and proposed a universal definition of health literacy: “individuals have the ability to obtain, understand and process basic health information or services, and can use information and services to promote individual health” [5]. In 2003, the American Association of Medical Libraries (MLA) combined the concepts of health literacy and information literacy. The concept of health information literacy was proposed for the first time, which is “the ability to identify the needs of health information, identify possible information sources and use them to retrieve relevant information; to evaluate the quality of information and its applicability to specific situations; to analyze, understand, and make good health decisions by using this information” [6], including the awareness of health information needs, the screening and acquisition of health information, analysis, evaluation, and use of healty information. Since then, health information literacy has been gradually incorporated into the research system of public health statuses. The early Chinese research on health information literacy mainly included reviews of international research, and most of its theoretical research that was a translation of international literature. In 2005, a health literacy survey was carried out in China, and a health literacy assessment system was established [7]. In 2012, health information literacy was included in the monitoring and evaluation of the residents’ health literacy for the first time in China [8]. In 2013, Wang et al. interpreted the connotations of health information literacy from the aspects of medical treatment, prevention, healthcare, and rehabilitation, and they pointed out that health information literacy is the comprehensive embodiment of an individual’s ability to perceive, identify, obtain, utilize, and creatively process health information and its value [9].

Based on comprehensive grasping the development of health information literacy and its connotations at home and abroad, in this paper, we focus on the concept of the development of health information literacy in the field of epidemic prevention and control, namely, epidemic prevention information literacy. From its origin, epidemic prevention information literacy has been the combination of epidemic prevention literacy and information literacy. Epidemic prevention information literacy is the derivation and development of health information literacy based on specific epidemic prevention situations and information environments. Epidemic prevention literacy is an important component of health literacy, involving the individual’s understanding and application of the occurrence, prevention, and treatment of diseases [10], as well as an individual’s comprehensive ability to acquire, evaluate, and use information resources to solve practical problems [11]. Therefore, we believe that epidemic prevention information literacy is the comprehensive embodiment of the awareness, attitude, and ability of the individual to understand, demand, obtain, evaluate, process, and apply epidemic prevention information and assess its value.

### 2.2. Research on Evaluation Tools and Contents of Epidemic Prevention Information Literacy

Internationally, epidemic prevention information literacy focuses on the individual access to epidemic prevention information, and their ability to evaluate and apply this information. Epidemic prevention information literacy is based on health information literacy, and it is a comprehensive application of health information literacy in epidemic prevention and control. Therefore, combing the research on health information literacy has important reference value for the evaluation framework and practice of epidemic prevention information literacy. The research on the health information literacy evaluations in developed countries in Europe and the United States started early and has rapidly developed. The related research can be divided into two aspects: (1) theoretical exploration, and (2) practical cultivation. In terms of theoretical research, referring to the theoretical framework proposed by Nutbeam, health information literacy can be divided into three categories: functional, interactive, and critical [12]. Functional health information literacy assessment tends to evaluate the basic literacy of clinical patients on the reading, understanding, and calculation of health-related medical terms and indicators, which mainly provides a reference for the effective evaluation of a community’s basic medical care and public health. Assessment plays an important and foundational role in the field of functional health information literacy. The representative tools are the “Rapid Estimate of Adult Literacy in Medicine (REALM)” [13] and the “Test of Functional Health Literacy in Adults (TOFHLA)” [14]. The REALM mainly tests the abilities of individuals to read and spell common medical terms, and the TOFHLA tests the calculation abilities of health-related data. Active health information literacy assessment is based on functional health information literacy, and it is mainly used to evaluate the cognitive literacy and social skills of individuals who obtain information from the media environment and apply new information to the context of their health needs [15]. The subjects have begun to extend from patients to ordinary citizens. The representative tools are the Research Readiness Self-Assessment (RRSA) and Everyday Health Information Literacy (EHIL) screening tool. The RRSA is used to evaluate the search, evaluation, and identification abilities of online health information of college students [16]. The EHIL [17] screening tool pays attention to the interaction abilities of individuals in the information society and environment, and it takes the interaction ability of health information needs, evaluation, and skills as the evaluation content. Critical health information literacy emphasizes the individual’s ability to accurately analyze, judge, and evaluate health information. At present, only the Communication and Critical Scale is used to evaluate the effective communication and critical thinking abilities of individuals.

In developed countries, the main method of cultivating public information literacy is consultation services. The National Institute of Health (NIH) and Medical Library Association (MLA) have played important roles in providing epidemic prevention information literacy education to the American public. Through the establishment of public health librarians and the opening of health information services for the public, the equity and accessibility of health services are ensured [18]. In addition, the National Library of Medicine (NLM) and National Health Service (NHS) of the United Kingdom have created a health information support service system that ensures that patients and citizens can obtain and correctly understand health information through free consultations and other services to promote the cultivation of epidemic prevention information literacy [19].

The research on epidemic prevention information literacy in China started relatively late, and there is currently no recognized authoritative scale. The health literacy monitoring released in 2012 included health information literacy in the monitoring scope for the first time; however, this was not the focus of the monitoring [20]. In 2013, the self-assessment scale for health information literacy [21] drew on the theory of health information literacy proposed by the American Medical Library Association (MLA), used self-assessment to examine individual self-esteem, divided residents’ health information literacy into health information awareness, health information acquisition, health information evaluation, health information application, health information morals, etc., and initially formed a network that integrated medical care, healthcare, and prevention. The health information literacy evaluation model that integrates rehabilitation provides a model reference for practical research on epidemic prevention information literacy. The 2014 health information literacy questionnaire [22] refers to the Everyday Health Information Literacy (EHIL) screening scale prepared by Finnish scholars, which assesses the ability of individuals to obtain, understand, and identify online health information; however, the assessment items are few and the content is not comprehensive. In the post-epidemic era, the college students’ health information literacy scale assesses the individual’s health information awareness, acquisition, knowledge, evaluation, application, and morality; however, the assessment content is also limited to the first-level dimension [23].

### 2.3. Research on Epidemic Prevention Information Literacy

Epidemic prevention information literacy is one of the most important components of health information literacy, and it is not only the focus of epidemic prevention situations based on health information literacy, but also the extension of the application of health information literacy. Therefore, based on the connotation and structure of health information literacy, epidemic prevention information literacy should highlight the information characteristics of epidemic transmission and construct the evaluation dimension under the structural framework of health information literacy. Generally speaking, although the public has been paying more and more attention to information literacy relevant to epidemic prevention, compared with the developed countries in Europe and the United States, the research on the evaluation of the information literacy of Chinese citizens is still relatively weak. The connotation of epidemic prevention information literacy is relatively vague, and authoritative evaluation tools are lacking. A small number of health information literacy evaluation studies focus on universal health information knowledge, ignoring the public’s concern with the ability to cope with the risk of epidemic diease. In addition, a practice system of the information literacy of national epidemic prevention has not been established, and research on the practice evaluation and cultivation of national epidemic prevention information literacy is scarce. Therefore, it is necessary to help citizens to realize the importance of epidemic prevention information literacy, establish a scientific and reasonable information literacy evaluation tool, and improve their abilities to obtain, understand, analyze, and use epidemic prevention information to make correct and timely epidemic prevention decisions.

## 3. Methodology

### 3.1. Construction and Interpretation of Evaluation Index System

This research starts with epidemic prevention literacy, refers to the main dimension and evaluation framework of health information literacy, pays attention to the important role that it plays in society, connotes the evolution and new requirements of the information era of epidemic prevention information literacy, and extracts and summarizes the core indicators of citizens’ epidemic prevention information literacy.

The evaluation index system of the epidemic prevention information literacy of Chinese citizens was preliminarily constructed with the help of thirteen experts in related fields. We considered that epidemic prevention information literacy consists of four first-class indexes and eleven second-class indexes, including epidemic prevention information awareness, epidemic prevention information knowledge, epidemic prevention information ability, and epidemic prevention information morality. Among them, “epidemic prevention information awareness” means that citizens realize the impact of epidemic prevention information on health, clarify their own needs for epidemic prevention information, and learn and share epidemic prevention information. Epidemic prevention information awareness includes the awareness of citizens that they need epidemic prevention information, that they can accurately and completely express their desire for it, and that they can consciously and actively obtain epidemic prevention information through reading, research, practice, and other learning methods, coordinate epidemic prevention information resources, and share epidemic prevention information with relevant personnel.

“Epidemic prevention information knowledge” refers to the basic and applied knowledge that citizens should reserve under different epidemic prevention scenarios. It includes the basic knowledge of epidemic prevention information, such as the basic theory, prevention and control methods, the epidemic development process, future trends, and how to apply the basic epidemic prevention knowledge to the actual epidemic prevention scene while considering their own health conditions.

“Epidemic prevention information capability” refers to the ability of citizens to effectively obtain, evaluate, and apply epidemic prevention information by using information equipment. It includes the ability of citizens to understand and use modern tools or software related to epidemic prevention information to obtain epidemic prevention information, as well as their ability to correctly understand the content of the epidemic prevention information, evaluate the authority and quality reliability of epidemic prevention information sources, and selectively integrate the epidemic prevention information into their own knowledge systems. There are three aspects to meet the actual health needs of epidemic prevention and control.

“Epidemic prevention information morality” refers to citizens’ cognition of and ability to respond to epidemic prevention morality, laws and regulations, and safety in the process of epidemic prevention and control. It includes the moral principles of epidemic prevention information that consciously abide by the code of conduct and moral standards recognized in epidemic prevention and control in the process of obtaining, using, processing, and disseminating epidemic prevention information, and it correctly handles the relationship between the development, dissemination, and use of epidemic prevention information. We should learn and abide by the relevant laws and regulations, such as the correct transmission of epidemic information and other relevant laws and regulations, master the safety knowledge of epidemic prevention information transmission, consciously maintain the safety of the epidemic information transmission information system, and reasonably use the epidemic prevention information in a healthy way.

### 3.2. Participants

During the outbreak of COVID-19, simple random sampling was used to conduct research. Participants filled in the questionnaire anonymously and independently. In total, 2736 valid samples were collected from 8 provinces and cities in China, and the effective recovery rate was 95.74%. Basic information: 1372 males and 1364 females; 2361 Han people and 375 other nationalities; 1429 urban residents and 1307 rural residents; 857 in the eastern region, 933 in the central region, and 946 in the western region; 656 minors (0–17 years old), 897 young people (18–40 years old), 1012 middle-aged people (41–65 years old), and 171 elderly people (over 65 years old); 1543 with basic education and 1193 with higher education.

### 3.3. Research Tool

The questionnaire was scored using a five-point Likert scale. The scoring standard was from 5 to 1, from “completely consistent” to “completely inconsistent”, respectively. Based on the evaluation index system, a test tool with high reliability and validity was developed. The overall Cronbach’s α was 0.86, including the Cronbach’s α of the epidemic prevention information awareness, epidemic prevention information knowledge, epidemic prevention information ability, and epidemic prevention information morality. The coefficients were 0.94, 0.91, 0.92, and 0.88, respectively, which indicated that the questionnaire had high reliability. In terms of validity, 13 experts in related fields were invited to revise and evaluate the consistency between the index system and test questionnaire, as well as the clarity of the expression content. Finally, the experts all observed that the key points reflected in the questionnaire were basically consistent with the index collection points. Moreover, a confirmatory factor analysis showed that the load factors of each factor were 0.70 and above, indicating that the test questionnaire had high validity.

### 3.4. Data Analysis

Half (1368) of the formal test data was randomly selected for the exploratory factor analysis, and the other half (1368) was used for the confirmatory factor analysis. Using SPSS 24.0 statistical software, principal component analysis, and maximum variance rotation analysis, the KMO sampling fitness measure was 0.93, and the significance of the Bartlett Sphericity test was less than 0.001, indicating that the scale was suitable for exploratory factor analysis. Factors with eigenvalues greater than 1 were extracted from 33 items, and 4 common factors were extracted after the orthogonal rotation of the factor load matrix. The cumulative variance interpretation rate was 63.03%. The load values of each item were between 0.71 and 0.86 on the corresponding factors, indicating that the load distribution was ideal. The four factors were basically consistent with the theoretical model of epidemic prevention information literacy constructed in this study. Adding to the factor score in the factor analysis, the proportions of the variance contribution rates of the four common factors to the total variance contribution rate were used as the weights to carry out the weighted calculation [24]. The weights of the four factors were as follows: 34.08%: epidemic prevention information ability (X_1_); 27.25%: epidemic prevention information awareness (X_2_); 22.49%: epidemic prevention information morality (X_3_); 16.18%: epidemic prevention information knowledge (X_4_), which means that the evaluation model of citizen epidemic prevention information literacy (Y) is as follows: Y = 0.34X_1_ + 0.27X_2_ + 0.23X_3_ + 0.16X_4_. Among the four dimensions of epidemic prevention information literacy, the contribution rate of the epidemic prevention information ability was the largest, and that of the epidemic prevention information knowledge was the smallest. The four dimensions complement each other and constitute the information literacy of citizens.

**Figure 1:**
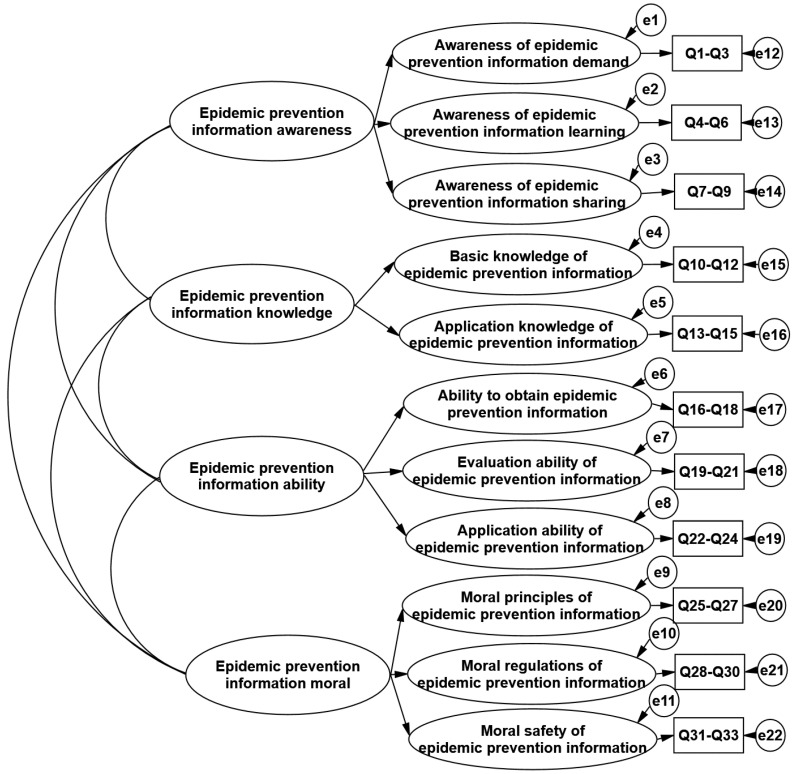
Second-order-model diagram of confirmatory factor analysis.

Confirmatory factor analysis (CFA) aims to determine the scale factor structure or model of a group of variables, and it generally includes model setting, model identification, a model parameter test, model evaluation, etc. Based on the theoretical analysis and exploratory factor analysis, in order to verify whether the four-dimensional model of citizen epidemic prevention information literacy was suitable for the actual data, confirmatory factor analysis was used to verify the structure of the formal questionnaire. A second-order model was proposed, as shown in Figure 1, and AMOS21.0 was used for the data analysis. As shown in Table 1, the standardization path coefficient of each secondary dimension was between 0.70 and 0.92, and the standardization factor load of each measurement index was between 0.70 and 0.93, indicating that each measurement index had high explanatory power and importance for its potential variables. The model goodness of fit was a direct evaluation of the rationality of the structural model, and it was mainly measured by a series of model fitting indexes (χ^2^/df = 4.57; RMSEA = 0.05; TLI = 0.92; CFI = 0.93), with all of them meeting the criteria of the discriminant analysis [25], which shows that the structural equation model of epidemic prevention information literacy had a good fit and fitting degree with the actual data.

## 4. Results

### 4.1. Overall Level of Epidemic Prevention Information Literacy of Citizens

To understand the current situation of the epidemic prevention information literacy of Chinese citizens, the model was preliminarily applied to analyze the current situation of 2736 random samples of data from 8 provinces in China. The results show that the overall score of the citizen epidemic prevention information literacy was 3.86, which indicates that the overall level of the epidemic prevention information literacy is high. In the four dimensions, the citizens’ awareness of epidemic prevention information (μ = 3.92, SD = 0.87) and epidemic prevention information knowledge (μ = 3.90, SD = 0.95) levels were relatively high; however, their epidemic prevention information ability (μ = 3.85, SD = 0.79) and epidemic prevention information moral (μ = 3.77, SD = 0.74) levels were relatively low.

### 4.2. Differences in Epidemic Prevention Information Literacy of Citizens

We observed significant differences in the levels of education and the locations of citizens in terms of their epidemic prevention information literacy (see Table 2). In terms of their epidemic prevention information awareness, the epidemic prevention information awareness of the citizens with higher education was significantly higher (t = −3.74, *p* < 0.001), the level of epidemic prevention information awareness of the rural residents was significantly higher than that of the urban residents (t = 4.71, *p* < 0.001), and the epidemic prevention information awareness of the citizens in the central and eastern regions (F = 59.01, *p* < 0.001) was significantly higher than that of the citizens of the western regions. In terms of their epidemic prevention information knowledge, the level of epidemic prevention information knowledge of the citizens with higher education was significantly higher (t = −11.47, *p* < 0.001), and the level of epidemic prevention information knowledge of the citizens in the central and eastern regions was significantly higher than that of the citizens in the western regions (F = 29.08, *p* < 0.001). In terms of their epidemic prevention information abilities, the information abilities of the citizens with higher education (t = −6.35, *p* < 0.001) was significantly higher those that of the citizens in the central and eastern regions (F = 18.63, *p* < 0.001). In terms of the epidemic prevention information moral, the level of epidemic prevention information morality of the rural citizens was significantly lower than that of the urban residents (t = −8.70, *p* < 0.001).

## 5. Discussion and Conclusions

### 5.1. Overall Level of Citizen Epidemic Prevention Information Literacy

Generally speaking, the level of the epidemic prevention information literacy of Chinese citizens is high; however, their ability to obtain, evaluate, and apply epidemic prevention information is low, and the moral of abiding by the law of epidemic prevention information dissemination is obviously insufficient. First of all, there is a certain distance between the information abilities of Chinese citizens and the requirements of the knowledge-based economy and society, which has led to the lack of information abilities of citizens in terms of access, evaluation, and application [26]. Moreover, the average cultural level of Chinese citizens is low, and so it is difficult for them to adapt to the diversified network epidemic prevention information retrieval and acquisition methods in the information age. Secondly, the moral level of citizens in terms of abiding by epidemic prevention information dissemination according to the law is relatively low. The main reason is that the moral education on citizens’ information literacy has always been a weak part in China’s education system [27], which has led to the weakness in and lack of citizen legal consciousness [28]. Moreover, the current laws and regulations on information dissemination in China are not perfect, and the enforcement is weak [29]. Therefore, in the process of spreading epidemic prevention information, the moral deficiency of citizens cannot be punished, and the problem of the moral deficiency in epidemic prevention information is more serious.

### 5.2. Information Literacy of Citizens and Differences in Various Dimensions

The awareness of epidemic prevention information is significantly affected by an individual’s education level and location. The epidemic prevention information awareness of the citizens with higher education was significantly higher. Previous studies have shown that health awareness is affected by education levels [30]. Citizens with higher education pay more attention to their own safety protection, their epidemic prevention information sources are rich, and they can obtain epidemic prevention information from the network, paper media, television, and interpersonal networks. Therefore, citizens with higher education levels have a greater awareness of epidemic prevention needs and learning awareness. In recent years, the health awareness of citizens in rural areas has been aroused and improved due to the modern rural medical and health service [31]. The awareness of citizens in the central and western regions was significantly higher than that in the eastern region. The average education level of the citizens in the eastern region was higher than those in the central and western regions, which has resulted in the lower epidemic prevention information awareness of the central and western residents’ than that of the eastern residents [32]. In addition, the form of disease prevention education in the central and western regions has not filtered deep into the society. In economically underdeveloped areas, citizens are affected by traditional feudal superstitions [33]. The distrust in modern epidemic prevention information has led to a large gap between the central and western regions of China and their eastern compatriots.

The knowledge of epidemic prevention information is significantly affected by an individual’s education level and region. Firstly, the knowledge level of epidemic prevention information of the citizens with higher education was higher than that of the citizens with only a basic education. Studies have shown that education level is an important factor that affects health literacy [34,35]. People with higher education levels usually have better knowledge reserves in terms of epidemic prevention, stronger abilities to obtain, understand, and apply health information, and more extensive access to health and epidemic prevention information [36]. Secondly, the knowledge level of epidemic prevention information in the eastern region was higher than those in the central and western regions. Epidemic prevention information knowledge is highly valued in eastern China, and the promotion of the daily health information in the rural areas of eastern China is as high as 80% [37]. Moreover, the economic and social development of the eastern region is fast, and many highly educated and talented people are gathered there. When facing a serious epidemic situation, they can quickly master the basic knowledge and application knowledge of epidemic prevention information, and their knowledge reserves are rich.

An individual’s epidemic prevention information ability is significantly affected by his/her education level and region. Firstly, the overall epidemic prevention literacy abilities of citizens with higher education is significantly higher than that of citizens with only a basic education. Citizens with high education levels have strong abilities to learn and master health skills. Secondly, the overall level of the epidemic prevention information capacity in the eastern region was higher than those in the central and western regions. The eastern region has better economic conditions, better access to information channels and conditions, a stronger influence of education, a higher information demand, and its citizens are better able to obtain information than those in the central and western regions. Thus, the information ability level of the citizens in the eastern region is higher.

The citizen epidemic prevention information morality is significantly affected by region. The epidemic prevention information morality of the citizens in urban areas was significantly higher than that of the citizens in rural areas. Firstly, rural citizens are less able to understand information, and their ability to obtain and screen information is low, which makes it more difficult for rural citizens to correctly use information [38]. Secondly, the average education level of rural citizens is lower than that of citizens of urban areas [39]. Therefore, it is difficult for rural citizens to abide by epidemic prevention information morality and correctly transmit epidemic prevention information. Finally, one of the main transmission channels of the epidemic prevention information of rural citizens is traditional interpersonal communication, during which they are easily misled and often blindly follow suit.

## 6. Suggestions

In this study, we constructed a scientific and reliable evaluation model of the epidemic prevention information literacy of Chinese citizens, and their information literacy was evaluated. The results show the following: (1) the epidemic prevention information abilities and epidemic prevention information morality of Chinese citizens are relatively low, and (2) the level of education and the location have a significant impact on the epidemic prevention information awareness, knowledge, ability, and moral of Chinese citizens. Based on this, to further cultivate and comprehensively improve the overall level of epidemic prevention information in China, the following countermeasures and suggestions are proposed.

### 6.1. Update Citizens’ Awareness and Concept of Epidemic Prevention Information, and Strengthen Publicity and Education for People in Vulnerable Areas

From the perspectives of the overall strategy of the prevention and control of public health emergencies and the cultivation of citizen safety and health literacy, we need to understand the significance of citizen information literacy in improving the public’s ability to respond to public health emergencies, and in the promotion of the modernization of the public health safety and governance. The thought is the guidance of action, and the renewal and transformation of consciousness, and the idea is the basis and guarantee to improve the information literacy of citizens. Additionally, publicity and guidance, community science popularization activities, and other means, will help the citizens in the central and western regions, and those with low education levels, to realize the important role of epidemic prevention information literacy, and to realize that, in the information society, epidemic prevention information literacy plays an important role in the prevention and control of major public health and safety incidents, and that it has risen to be a decisive factor in improving public epidemic prevention abilities of citizens, gradually helping them to establish a scientific and reasonable awareness and concept of epidemic prevention information.

### 6.2. Establish a Perfect Epidemic Information Service Platform and Speed Up the Education of Scientific Epidemic Prevention Knowledge

We need to promote the in-depth integration of information technology and the improvement in citizen epidemic prevention information literacy, as well as give full play to the role of new media in public epidemic prevention information literacy education. Moreover, we need to integrate information resources related to epidemic prevention and health, build a national authoritative network platform, release timely, authoritative, and professional epidemic prevention information and knowledge, clarify fallacies, and respond to people’s livelihood concerns. Furthermore, we need to speed up the training of citizens in the central and western regions in China in the basic knowledge of modern information science and new information technology, as well as basic safety and health knowledge, to help them adhere to rationality, respect science, guard common sense, rationally and scientifically analyze and screen information, and use authoritative information for guidance in scientific epidemic prevention and protection.

### 6.3. Deepen Coordination and Linkage of Multiple Departments and Jointly Promote Improvement in Citizen Epidemic Prevention Information Abilities

We should formulate relevant policy documents as soon as possible to provide an effective guarantee at the policy level for the cultivation of epidemic prevention information abilities of citizens. The education to improve epidemic prevention information abilities of citizens involves not only public health management departments, but also education and information departments. The State Council should jointly develop and issue training programs to enhance the epidemic prevention information capabilities of citizens as soon as possible to jointly promote the improvement in their epidemic prevention information abilities. Additionally, we should give full play to the education and publicity power of nongovernmental organizations, as well as carry out educational series of lectures, consultations, and other activities through schools, communities, public libraries, and other organizations to enhance the judgment of citizens in their analysis of information value, help them establish scientific prevention and control concepts, and enhance their rationality and judgment.

### 6.4. Strengthen Publicity of Moral Laws and Regulations, and Implement Safe Dissemination of Epidemic Prevention Information According to Law

We need to enhance the moral level of rural citizens by preaching information and strengthening the moral education in rural areas. Furthermore, we should improve the network information policies and regulations, establish a sustainable and executable information moral and legal system, and regulate and restrict the anomie behavior of epidemic prevention information dissemination.

This study provides a set of methods and norms for the evaluation of the information literacy of citizens in the post-epidemic era. In terms of specific content, the construction of the evaluation indicators was first based on the theoretical understanding of the evaluation framework related to health information literacy, using the literature research method and comparative research method to clarify the connotations of citizen epidemic prevention information literacy, and at the same time, we constructed a theoretical model and carried out empirical tests. At the practical level, the results of this study provide a reference for improving citizen epidemic prevention information literacy, and they also provide a tool to support the establishment of the evaluation norm of epidemic prevention information literacy. In the post-epidemic era, with the deepening of the research on epidemic prevention information literacy and health information literacy, the development path and training mechanism of citizen epidemic prevention information literacy are also constantly being improved. In follow-up related research, the team will start from big data research to build the norm of citizen epidemic prevention information literacy to provide reference for relevant research and education practice.

## Figures and Tables

**Table 1:** Factor load matrix of second-order model.

Secondary Dimension	Standardized Path Coefficient	Item Quantity	Standardized Factor Load		
Awareness of epidemic prevention information demand	0.92	3	0.88	0.93	0.86
Awareness of epidemic prevention information learning	0.86	3	0.81	0.87	0.88
Awareness of epidemic prevention information sharing	0.71	3	0.88	0.83	0.70
Basic knowledge of epidemic prevention information	0.91	3	0.73	0.72	0.78
Application knowledge of epidemic prevention information	0.88	3	0.84	0.81	0.85
Ability to obtain epidemic prevention information	0.70	3	0.81	0.77	0.80
Evaluation ability of epidemic prevention information	0.70	3	0.76	0.75	0.73
Application ability of epidemic prevention information	0.76	3	0.78	0.77	0.71
Moral principles of epidemic prevention information	0.74	3	0.88	0.70	0.78
Moral regulations of epidemic prevention information	0.81	3	0.72	0.72	0.73
Moral safety of epidemic prevention information	0.81	3	0.72	0.73	0.76

**Table 2:** Heterogeneous test in different dimensions of epidemic prevention information literacy.

Background Variables		Awareness of Epidemic Prevention Information (M ± SD)	Epidemic Prevention Information Knowledge (M ± SD)	Epidemic Prevention Information Capability (M ± SD)	Epidemic Prevention Information Moral (M ± SD)
Education level	Elementary education	3.86 ± 0.83	3.73 ± 0.99	3.77 ± 0.78	3.77 ± 0.72
	Higher Education	3.99 ± 0.92	4.13 ± 0.84	3.96 ± 0.78	3.78 ± 0.77
	t	−3.74 ***	−11.47 ***	−6.35 ***	−0.06
Census register	Countryside	4.00 ± 0.86	3.90 ± 0.95	3.88 ± 0.79	3.65 ± 0.79
	Town	3.84 ± 0.88	3.90 ± 0.95	3.82 ± 0.78	3.89 ± 0.68
	t	4.71 ***	0.01	1.96	−8.70 ***
Region	East ➀	4.11 ± 0.82	4.04 ± 0.88	3.98 ± 0.75	3.77 ± 0.82
	Central ➁	3.98 ± 0.67	3.96 ± 0.79	3.84 ± 0.68	3.80 ± 0.67
	West ➂	3.68 ± 1.03	3.72 ± 1.10	3.75 ± 0.89	3.75 ± 0.73
	F	59.01 ***	29.08 ***	18.63 ***	1.01
	LSD multiple comparison	➀ > ➁ > ➂	➀ > ➂, ➁ > ➂	➀ > ➁ > ➂	\

Note: *** *p* < 0.001.

## Data Availability

The data presented in this study are available on request from the corresponding author.
